# Measuring perceived racism and psychosis in African-Caribbean patients in the United Kingdom: the modified perceived racism scale

**DOI:** 10.1186/1745-0179-5-10

**Published:** 2009-05-20

**Authors:** Apu T Chakraborty, Kwame McKenzie, Gerard Leavey, Michael King

**Affiliations:** 1 Department of Mental Health Sciences, Royal Free & University College Medical School, Hampstead Campus, London, UK; 2 Social Equity and Health Research, Centre for Addiction and Mental Health, 455 Spadina Avenue, Suite 300, Toronto, Ontario, Canada; 3 Northern Ireland Association for Mental Health (NIAMH), Central Office, 80 University St, Belfast, UK

## Abstract

**Aim:**

The increased rate of psychosis and poorer service-related outcomes in UK African-Caribbeans may in part be related to racism; racism as an aetiological factor remains comparatively under-investigated. We wanted to develop a measure of perceived racism in UK African-Caribbean patients with psychosis

**Methods:**

We modified the Perceived Racism Scale (PRS) by substituting a mental-health-services' racism domain for the employment-racism domain and administered it to a sample of 150 individuals.

**Results:**

110 people completed the PRS with a total mean perceived racism score of 54.2 for the previous year and 71.3 for the lifetime. The modified instrument had good internal consistency, and both a similar factor-analytic structure and sampling adequacy to the original instrument.

**Clinical Implications:**

The modified PRS was acceptable to the sample, withstands statistical scrutiny and produced similar totals to those in previously-tested populations. Subjective measurement of perceived racism may improve understanding of psychosis in African-Caribbeans, improve engagement and, hopefully, outcome.

## Introduction

People of African-Caribbean origin with psychosis in the UK are reported to have different service-related outcomes and increased rates of psychosis relative to their white counterparts [1-3]. A literature-review indicates that both the excess risk of psychosis as well as the altered service-related outcome in UK African-Caribbeans with psychosis may at least in part be explained by racism [4].

There is limited work upon the association between racism and psychosis. A cross-sectional survey from a nationally representative UK community sample (EMPIRIC) found that perceived societal and interpersonal racism both exerted independent effects on the risk of psychosis in minority ethnic groups [5]. A population-based incidence and case-control study of first-episode psychosis (ÆSOP) similarly found that perceived disadvantage at least partly associates with the excess of psychosis among UK Black people [6]. A Netherlands group found a dose-response relationship between discrimination and incidence rate ratios of psychosis in ethnic minority groups [7]. However, their second study failed to find perceived discrimination to be a risk factor for schizophrenia [8]. Earlier UK work reported raised incidences of schizophrenia in all minority ethnic groups presenting to psychiatric services within a deprived, inner-city setting [9].

In order to establish an association between racism and psychosis, it is necessary to find an effective method to measure racism. Surveys provide an indirect measure of racism as they focus on self-reported attitudes rather than verifiable events. One such self-report instrument is the Perceived Racism Scale (PRS), which is a multi-dimensional measure of racism (contextual, attitudinal, behavioural and cultural) in African-Americans [10]. The PRS not only provides a measure of the frequency of exposure to many manifestations of racism, but takes a step forward by assessing emotional and behavioral coping responses to racism. These responses are measured in four situational domains: on the job, in academic settings, in the public realm, and on exposure to racist statements. That the PRS is subjective does not necessarily limit its validity. The subjective reality of individual patients may influence their engagement (and indeed outcome) on an individual level, which is why so many researchers call for researchers and service-providers to take on board what the patient is saying [11].

Cultural and institutional exclusion and racism have been reported to characterise the African-Caribbean community's experience of mental health services, discouraging people from approaching services [12]. To then expect participants to openly discuss sensitive areas like racism and psychosis with researchers may be perceived as unrealistic. However, the readiness with which patients would discuss and explore racism may demonstrate that it is an issue that they want to talk about [13]. Such issues could be dealt with in the consultation or in therapy depending on whether it is seen as a live issue in the individual's context. Sensitive exploration of these issues could be used to forge links between patients and their clinicians, perhaps improving the therapeutic alliance and outcome.

We set out to improve upon the measurement of perceived racism in minority ethnic groups in receipt of mental health services by modifying the Perceived Racism Scale and administering this to a group of African-Caribbean patients with psychosis.

## Method

### Study participants

Participants included in the study were aged above 18 years; had an ethnicity of "African-Caribbean origin", with either or both parents or grand-parents born on a Caribbean island; having a Research Diagnostic Criteria-defined psychotic illness (either schizophrenia or schizo-affective disorder); and in receipt of local mental health services. Those patients with a primary diagnosis of either an organic disorder or a substance-misuse disorder were excluded. Community Mental Health Team (CMHT) managers and Consultant psychiatrists for all of the CMHTs in Haringey and north Camden, London were asked to identify appropriate participants: a sample of 110 were then interviewed.

### Measuring perceived racism

The Perceived Racism Scale (PRS) was modified to measure racism. The PRS is an American, self-completed instrument that measures the frequency of exposure to perceived racism, emotions, coping behaviours, and cognitive appraisals related to the racist encounter. It explores four domains: at work, in academic settings, in the public realm (or everyday racism), and responses to racist statements.

We modified the PRS to make it more appropriate (see Additional file 1). Firstly, "Americanisms" were changed; secondly, domain statements pertaining to racism from mental health services replaced the work-domain statements, as it was considered more important to tap into racist experiences within mental health services than within the work-setting in this group. The new statements were developed using a number of sources: the first was a qualitative pilot study [14], which examined perceived racism of black African and African-Caribbeans with psychosis. The second source was a focus group with an early intervention team for young African and African-Caribbean patients with psychosis. The group critically and candidly reported the experiences and perceptions of racism within mental health services as described by their patients. The final source was the research-group's consensus, with academic and clinical experience in mental health service-provision for ethnic minorities in the UK.

When comparing the previous domain (work) and the new domain (mental health services), the subject content although naturally different, the wording and style of each statement remains relatively preserved. This was in order to maintain the integrity of the original PRS instrument as much as possible. The new domain was then piloted on a group of patients to test its acceptability and readability. The domain was found to be both appropriate and "user-friendly" and thus was incorporated into the PRS.

The rest of the assessment consisted of: a socio-demographic assessment; the World Health Organisation Life-Chart [15], the Structured Clinical Interview – Positive and Negative Syndrome Scale [16], the modified Perceived Racism Scale (primary exposure), and the Operational criteria checklist for psychotic and affective illness [17].

### Statistical comparison with original Perceived Racism Scale

We compared the statistical functionality of the modified PRS against the original PRS [10], using the derived data. Internal reliability of both the mental health services domain alone and the complete scale were compared using Cronbach's Coefficient Alpha. Items from the scale were divided into two portions based on question type: (a) frequency of exposure, questions 1–43; and (b) emotional and coping responses, questions 44–51 (68 items). Principal component factor analyses were conducted on the frequency of exposure items using first an orthogonal (varimax) and then an oblique (promax) rotation [there were insufficient observations to conduct these on the emotional and coping responses]. We obtained Eigenvalues, then visualised on a scree plot to compare the magnitude of the most significant factors against the original scale. Finally, Kaiser's measurement of sampling accuracy was performed for the frequency of exposure questions to allow a further comparison.

## Results

Socio-demographic and clinical data for the sample (n = 110) is given: 55% of the sample was male and the average age was 43 years, ranging from 18 to 79 years. Participants' fathers born in the Caribbean formed the largest group (86.4%). The majority of participants were single (80%) and unemployed (97.3%). Most participants had an OPCRIT-generated ICD-10 diagnosis of schizophrenia (72.7%) and most were hospital in-patients (49.1%). The greatest proportion of participants had moderately severe symptoms (51.8%) and most had a continuous-type illness (48.2%). Approximately one-third of participants had had at least one hospital admission (36.4%) and all had been prescribed anti-psychotic medication.

Internal consistency of the new domain was determined using Cronbach's Coefficient Alpha and compared with McNeilly's original study [10] and is detailed in Table 1. As can be seen, the values are both high and similar in magnitude.

**Table 1:** Internal reliability for modified perceived racism scale (PRS) in comparison with original PRS (Cronbach's alpha calculated across domains)

	Alphas for combined student and community US sample (n = 214)	Alphas for student US sample (n = 110)	Alphas for community US sample (n = 104)	Alphas for UK sample (n = 110)
Frequency of exposure domains (pooled)	0.96			0.96

Frequency of exposure subscale responses (job/mental health services, over past year)	-	0.88	0.92	0.87

Frequency of exposure subscale responses (job/mental health services, over life)	-	0.92	0.93	0.85

Emotional responses (aggregated)	-	0.94	0.94	0.91

Next, a similar factor analysis to McNeilly's study was executed to permit comparison and the results are given in Table 2. Principal component factor analysis was carried out on the frequency of exposure items using first orthogonal, then oblique rotations. Both orthogonal and oblique rotations produced nearly identical factor sets and so, as in the original work, values for the orthogonal rotation are presented. Similarly, factor rotations for "a" ("over the past year") and "b" ("over my life") for the frequency of exposure items were nearly identical. Therefore, only values for "a" responses are presented to enable comparison. Factor loadings of the new domain were almost all above 0.5 which was acceptable; they were similar in magnitude to the original data, also. The coefficient alphas for both old and new domains were similarly high in value, with the new domain accounting for relatively more variance (20%) than the old domain (6%).

**Table 2:** Principal components factor analysis (orthogonal rotation) of items measuring frequency of exposure to perceived racism; comparison with original PRS

Factor 1: Frequency of Exposure to Racism on the Job/Mental Health Services	Item loading (Job)	Item loading (Mental Health Services)	Variance (Job)	Variance (Mental Health Services)	Cronbach's Coefficient Alpha (Job)	Cronbach's Coefficient Alpha (Mental Health Services)
Item Number			6%	20%	0.91	0.92

One	0.6318	0.4708				

Two	0.6523	0.5791				

Three	0.6948	0.6450				

Four	0.6951	0.5900				

Six	0.6475	0.3205				

Seven	0.6232	0.4753				

Eight	0.6561	0.6206				

Nine	0.6232	0.5257				

Ten	0.5766	0.5433				

Scree plots based on Eigenvalues suggested five factors from the pooled items concerning frequency of exposure, as in the original study. Again, Eigenvalues for all five factors were greater than one (ranging from 10.08 to 1.61). Finally, Kaiser's measurement of sampling adequacy yielded an overall 0.93 for the frequency of exposure items in the original work and 0.7910 in our study; values greater than 0.80 are considered good, and those less than 0.50, inadequate.

Table 3 shows the sample total-scores for perceived racism as determined by the Perceived Racism Scale (PRS), and in comparison to other studies. As can be seen, the figures obtained are broadly similar. In addition, total and individual domain PRS scores within our sample group were found to be stable irrespective of demographic characteristics: the scores did not vary significantly according to either age, gender or psychiatric diagnosis.

**Table 3:** Total perceived racism scale (PRS) scores for the sample and other studies

PRS domain score	Total racism, past year (range 0–210); Mean (S.E.)	Total racism, lifetime (range 0–230): Mean (S.E.)
Our study (n = 110)	54.2(1.84)	71.3(2.10)

Combs et al (n = 128)	49.9	

Steffen et al (n = 69)		50.75

Clark (n = 39)	75.25	

## Discussion

The modified Perceived Racism Scale (PRS) was acceptable to, and completed in full by, this sample of 110 African-Caribbean patients with psychosis. The statistical evaluation of the new domain showed it to be equivalent to the unmodified PRS. And the total modified PRS scores were similar to those obtained in other studies.

The study had limitations. It is unclear whether the group that agreed differed consistently to those who did not agree; the only information collected on those who did not participate indicated that they were more likely to be male and younger in age than participants. This may reflect the more general level of dissatisfaction with mental health services that has been reported in second- and third-generation African-Caribbeans when compared with their older counterparts [18]. Difficulties associated with self-report instruments such as the PRS are that of social desirability and bias created by the subject's perception of the researcher's ethnicity [19]. Another potential problem is that of the biased recall and overemphasis of adverse or memorable events. Related to this is the period effect when the predominant racial climate of the respondent's nation influences, either positively or negatively, the recall of racially related events.

The new mental health services domain performed well under statistical scrutiny: it showed good internal consistency, produced a similar factor profile to the original instrument and there was good evidence of post-estimation sampling adequacy.

This is the first known report of the measurement of perceived racism using a form of the Perceived Racism Scale (PRS) in a UK population with psychosis, which limits the ability to perform direct comparisons. However the original PRS has been used in US studies. The mean total scores obtained in this study were 54.2 for racism over the preceding year, and 71.3 for racism over a lifetime, with a higher score indicating more perceived racist events.

In a study of perceived racism and paranoia in African-American college students, the mean total PRS one-year score was remarkably similar [20]. A second study examined perceived racism and ambulatory blood pressure measured during daily life in employed African-Americans in the Duke Biobehavioral Investigation of Hypertension study [21]. The mean total PRS-lifetime score was considerably lower than our study. A third study examined the relationship between perceived inter-ethnic group racism and blood pressure responses in a group of African American college women [22], and the mean total PRS one-year score was found to be 75.25, significantly higher than our study.

The different scores across studies is hardly surprising given the different profiles of the sample populations in terms of geography, the presence of severe mental illness, socio-economic status, educational level, medication and tobacco use and so on. What is perhaps more interesting is the degree of similarity that appears to exist between the scores, with the greatest difference being no more than 10%. This may support a universality of the reporting of racism (and its measurement by the PRS) by those of African-Caribbean origin, irrespective of their backgrounds, cultural experiences and personal histories and this also speaks to the construct validity of the PRS.

## Competing interests

The authors declare that they have no competing interests.

## Authors' contributions

AC conceived of the study, collected and analysed the data and wrote the draft. KM assisted with the study's design and provided supervision. GL participated in the study's design, assisted with sourcing data and provided research facilities. MK led on the study's design and co-ordination, and gave mentorship. All authors contributed to drafts and approved the final manuscript.

## Supplementary Material

Additional file 1 The modified perceived racism scale. Click here for file
