# Supplementary material for: Measuring perceived racism and psychosis in African-Caribbean patients in the United Kingdom: the modified perceived racism scale
Source: Clin Pract Epidemiol Ment Health. 2009 May 20;5:10. doi: 10.1186/1745-0179-5-10 (PMC2692844; doi:10.1186/1745-0179-5-10)
Supplement: Additional file 1 — The modified perceived racism scale. [file 1745-0179-5-10-S1.doc]

**Additional file 1**

**Modified Perceived Racism Scale – Mental health services domain**

**Original Perceived Racism Scale – Employment domain**

**A. RACISM ON THE JOB:**

| 1. | Because I am Black, I'm assigned the jobs no one else wants  to do. | Not Applicable | Almost Never | Several Times  A Year | Several Times  A Month | Several Times  A Week | Several Times  A Day |
| --- | --- | --- | --- | --- | --- | --- | --- |
|  | a. How often has this happened in the past year? | 0 | 1 | 2 | 3 | 4 | 5 |
|  | b. How often has this happened during my life? | 0 | 1 | 2 | 3 | 4 | 5 |
|  |  |  |  |  |  |  |  |
| 2. | At work, when different opinions would be helpful, my  opinion is not asked for because of my race. |  |  |  |  |  |  |
|  | a. How often has this happened in the past year? | 0 | 1 | 2 | 3 | 4 | 5 |
|  | b. How often has this happened during my life? | 0 | 1 | 2 | 3 | 4 | 5 |
|  |  |  |  |  |  |  |  |
| 3. | I am treated with less dignity and respect than I would be if I were white. |  |  |  |  |  |  |
|  | a. How often has this happened in the past year? | 0 | 1 | 2 | 3 | 4 | 5 |
|  | b. How often has this happened during my life? | 0 | 1 | 2 | 3 | 4 | 5 |
|  |  |  |  |  |  |  |  |
| 4. | I am watched more closely than other workers because of my race. |  |  |  |  |  |  |
|  | a. How often has this happened in the past year? | 0 | 1 | 2 | 3 | 4 | 5 |
|  | b. How often has this happened during my life? | 0 | 1 | 2 | 3 | 4 | 5 |
|  |  |  |  |  |  |  |  |
| 5. | Racial jokes or harassment are directed at me at work. |  |  |  |  |  |  |
|  | a. How often has this happened in the past year? | 0 | 1 | 2 | 3 | 4 | 5 |
|  | b. How often has this happened during my life? | 0 | 1 | 2 | 3 | 4 | 5 |
|  |  |  |  |  |  |  |  |
| 6. | Because I am Black, I feel as if I have to work twice as hard. |  |  |  |  |  |  |
|  | a. How often has this happened in the past year? | 0 | 1 | 2 | 3 | 4 | 5 |
|  | b. How often has this happened during my life? | 0 | 1 | 2 | 3 | 4 | 5 |
|  |  |  |  |  |  |  |  |
| 7. | Tasks that require intelligence are usually given to Whites, while Blacks get those that don't require much thought. | Not Applicable | Almost Never | Several Times  A Year | Several Times  A Month | Several Times  A Week | Several Times  A Day |
|  | a. How often has this happened in the past year? | 0 | 1 | 2 | 3 | 4 | 5 |
|  | b. How often has this happened during my life? | 0 | 1 | 2 | 3 | 4 | 5 |
|  |  |  |  |  |  |  |  |
| 8. | I am often ignored or not taken seriously by my boss because of my race. |  |  |  |  |  |  |
|  | a. How often has this happened in the past year? | 0 | 1 | 2 | 3 | 4 | 5 |
|  | b. How often has this happened during my life? | 0 | 1 | 2 | 3 | 4 | 5 |
|  |  |  |  |  |  |  |  |
| 9. | Whites often assume I work in a lower status job than I do and treat me as such. |  |  |  |  |  |  |
|  | a. How often has this happened in the past year? | 0 | 1 | 2 | 3 | 4 | 5 |
|  | b. How often has this happened during my life? | 0 | 1 | 2 | 3 | 4 | 5 |
|  |  |  |  |  |  |  |  |
| 10. | A white co-worker with less experience and qualifications got promoted before me. |  |  |  |  |  |  |
|  | a. How often has this happened in the past year? | 0 | 1 | 2 | 3 | 4 | 5 |
|  | b. How often has this happened during my life? | 0 | 1 | 2 | 3 | 4 | 5 |
